# Successful Respiratory Management Using Synchronized Nasal Intermittent Positive Pressure Ventilation for Abnormal Breath Patterns Associated With Joubert Syndrome

**DOI:** 10.7759/cureus.99213

**Published:** 2025-12-14

**Authors:** Akihiro Managi, Masashi Zuiki, Rika Mitsuno, Eisuke Ichise, Tomoko Iehara

**Affiliations:** 1 Department of Pediatrics, Kyoto Prefectural University of Medicine, Kyoto, JPN

**Keywords:** apnea, joubert syndrome, molar tooth sign, synchronized nasal intermittent positive pressure ventilation, tachypnea

## Abstract

Patients diagnosed with Joubert syndrome (JBS) frequently present with differing respiratory irregularities, including tachypnea followed by apnea, particularly during infancy. Consequently, it is essential to adopt respiratory support strategies tailored to atypical breathing patterns associated with high mortalities. Herein, we report a case of successful respiratory management of abnormal breathing patterns associated with JBS based on synchronized nasal intermittent positive pressure ventilation (SNIPPV) using the MEdiTRIG (Medin Medical Innovations GmbH, Puchheim, Germany) pressure trigger system. A full-term male newborn presented with median cleft lip, abnormal palate with lingual hamartoma, and bilateral polydactyly. Brain magnetic resonance imaging revealed the characteristic molar tooth sign, and subsequent genetic testing identified a pathogenic splice-site variant of the OFD1 gene (c.2387+1G>T), confirming the diagnosis of JBS. Although respiratory management was initially established using bilevel non-invasive positive pressure ventilation, frequent episodes of apnea were observed. However, after transitioning to SNIPPV using the MEdiTRIG pressure trigger system at eight days of life, the frequency of apnea episodes was markedly reduced. This respiratory support enables high synchronization without requiring a conventional ventilator or additional catheter insertion. Our findings indicate that this system is effective for managing neonatal conditions characterized by differing degrees of respiratory disorders, such as JBS, leading to improved clinical outcomes for this patient population.

## Introduction

Joubert syndrome (JBS) was initially identified in 1969 by Joubert et al. as “familial agenesis of the cerebellar vermis,” characterized by cerebellar and brainstem dysfunction resulting from a partial or complete absence of the cerebellar vermis [1]. Clinically, JBS is distinguished by the molar tooth sign observed on brain imaging, which indicates cerebellar vermis deficiency and brainstem malformations [2,3]. Patients with this syndrome present with delayed psychomotor development, eye movement abnormalities, and gradual damage to the retina, kidneys, and liver. In addition, JBS is a clinically and genetically heterogeneous ciliopathy that presents with multi-system involvement. To date, at least 35 JBS subtypes have been classified based on causative genes, among which, JBS10 has been attributed to mutations in the OFD1 gene that encodes a protein essential for the development of primary cilia and the establishment of left-right asymmetry [4].

Patients diagnosed with JBS frequently present with respiratory abnormalities. Respiratory variability is a characteristic feature of this syndrome, as initially documented by Joubert et al., who described cases of a cerebellar disorder that included episodic tachypnea. Subsequently, Boltshauser and Isner reported three neonates with JBS who were characterized by rapid shallow breathing interspersed with periods of apnea [5]. Respiratory irregularities in JBS, including tachypnea followed by apnea, are particularly evident during infancy. Nevertheless, these conditions are challenging to manage with non-invasive positive-pressure ventilation, which is commonly employed in a neonatal intensive care unit. Furthermore, this respiratory pattern is associated with adverse neonatal outcomes, including mortality [6]. However, despite the imperative of implementing respiratory support strategies tailored to atypical breathing patterns in neonates, documentation of respiratory management specifically designed to address the abnormal breathing patterns in JBS remains limited.

Synchronized nasal intermittent positive pressure ventilation (SNIPPV) is a non-invasive positive pressure type of ventilation that provides biphasic positive pressure in synchrony with an infant's spontaneous breathing [7]. Furthermore, by detecting each breath and providing corresponding inspiratory and expiratory pressures, SNIPPV enables ventilatory assistance comparable to that obtained using mechanical ventilation, whilst remaining non-invasive. The MediTRIG pressure trigger system (Medin Medical Innovations GmbH, Puchheim, Germany), which has been specifically developed for premature infants and newborns, facilitates highly precise synchronization of this ventilation mode [8,9]. Herein, we report the first documented case of successful respiratory management using SNIPPV with the MEdiTRIG pressure trigger system for rectifying the abnormal breathing patterns associated with JBS.

## Case presentation

The patient was a male infant born at 37 weeks and one day of gestation via cesarean section, indicated for cephalopelvic disproportion. His family history revealed two healthy elderly sisters, with no congenital anomalies being identified on either parental side. Although on the maternal side, the mother's brother was found to have syndactyly of the fifth toe, and the maternal grandmother's brother had a cleft lip, there was no family history of JBS. Immediately following birth, an absence of initial crying, along with hypotonia, grunting, chest retraction, and nasal flaring were noted, and Apgar scores [10] at one, five, and 10 min were three, seven, and nine points, respectively.

**Figure 1:**
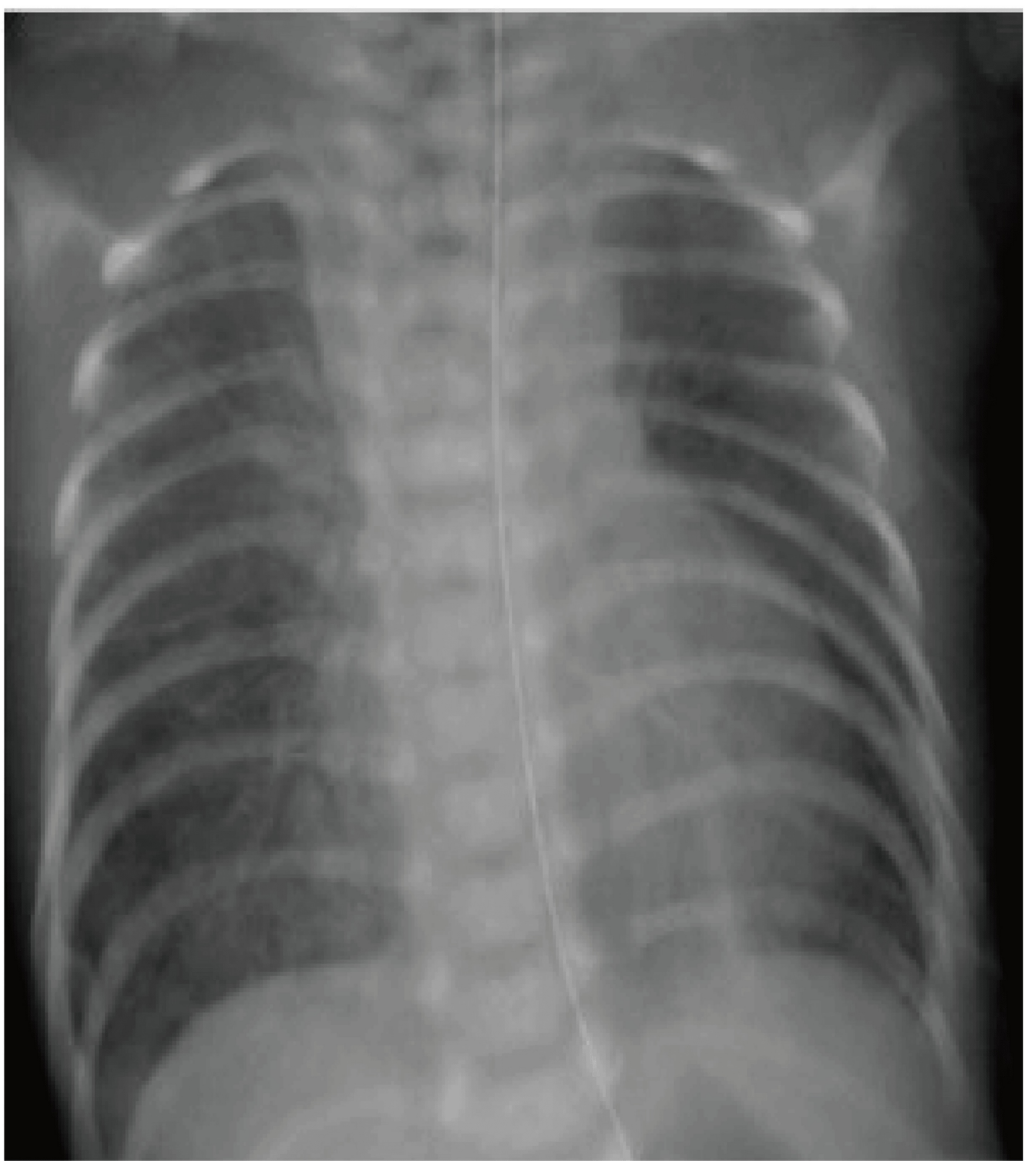
Postnatal chest radiography.

His birth weight was 2.782 g (±0 SD), body length was 49.5 cm (+1.0 SD), and head circumference was 36.0 cm (+2.5 SD). Physical examination revealed a median cleft lip, an abnormal palate with lingual hamartoma, bilateral polydactyly, and a 0.5 cm occipital mass. Breath sounds were bilaterally clear, and heart sounds were regular without murmurs. Postnatal chest radiography revealed reduced bilateral lung transparency (Figure 1).

**Figure 2:**
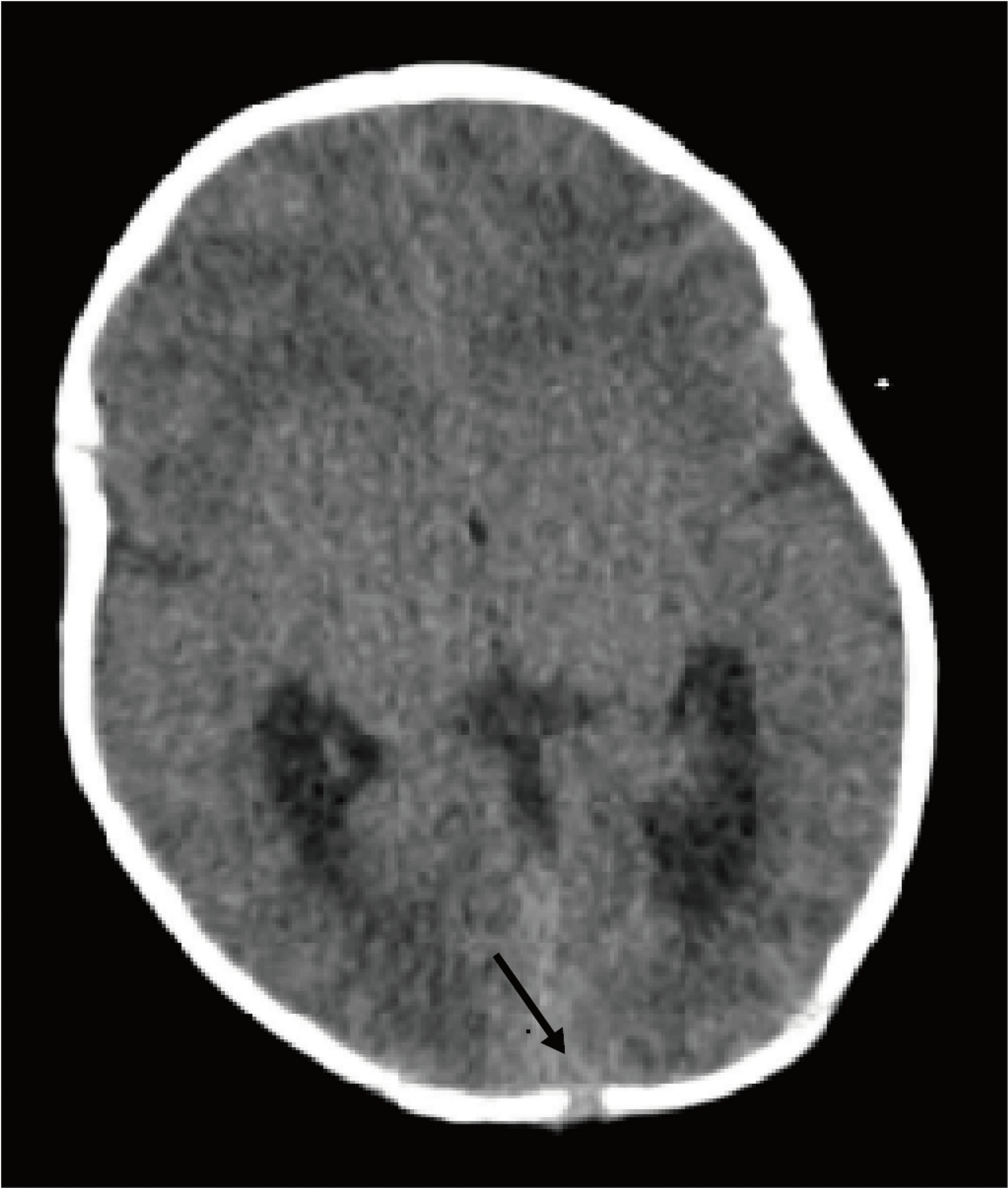
Axial computed tomography of the head revealed an encephalocele with intracranial communication.

Computed tomography of the head, performed on day 32, revealed an encephalocele with intracranial communication (Figure 2).

**Figure 3:**
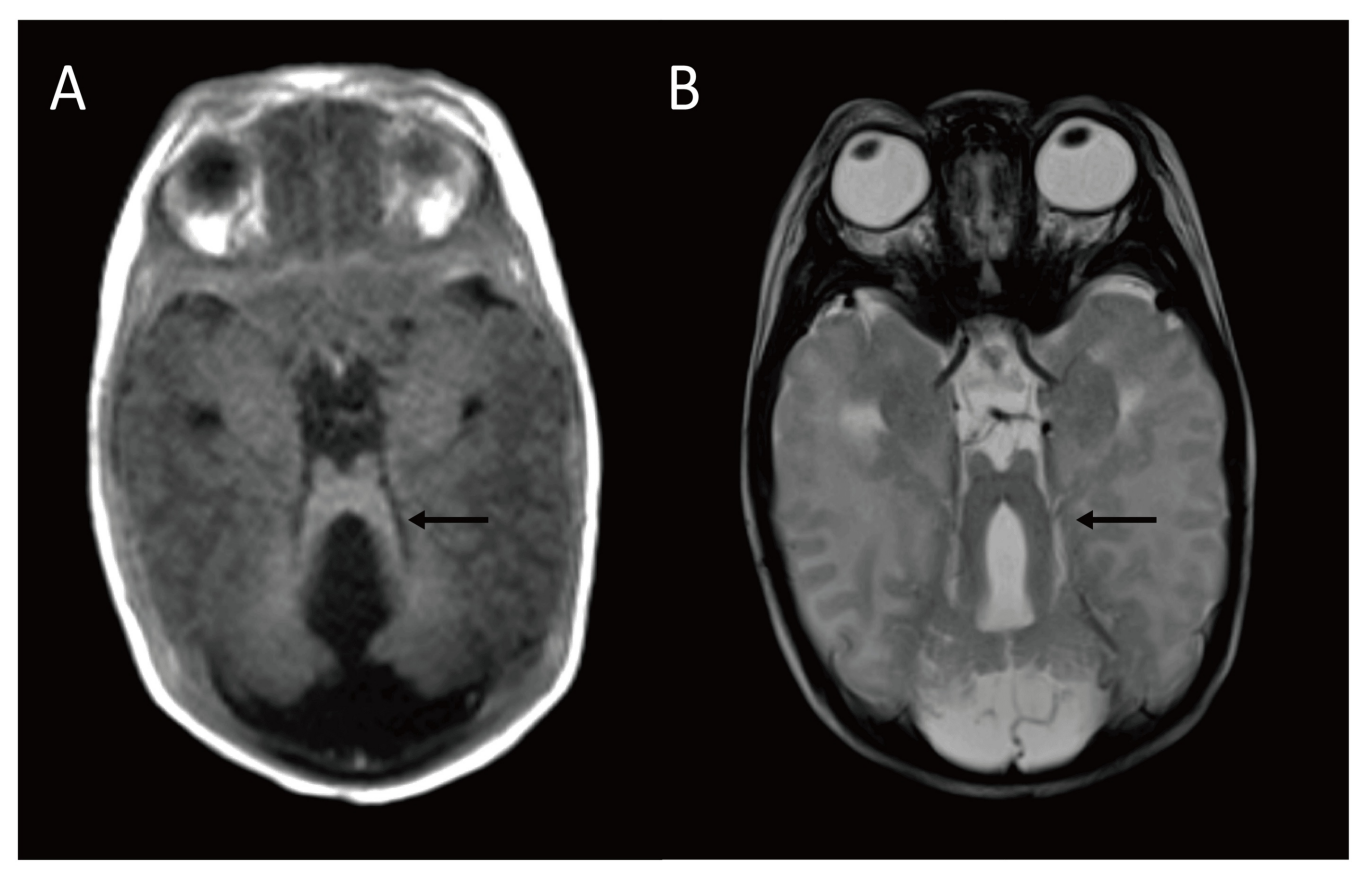
Magnetic resonance imaging of the brain revealed the molar tooth sign. (A) Axial T1-weighted image; (B) axial T2-weighted image.

In addition, magnetic resonance imaging of the brain on day 35 revealed an unusually deep interpeduncular fossa, thickened superior cerebellar peduncles, and agenesis of the cerebellar vermis, consistent with the molar tooth sign (Figure 3).

On the basis of these findings, a clinical diagnosis of JBS was established. Subsequently, a recently reported novel pathogenic splice-site variant of the OFD1 gene (c.2387+1G>T) that causes JSB10 was identified in this patient [11].

Following admission to the neonatal intensive care unit, non-invasive respiratory support was commenced using an Infant Flow™ SiPAP^®^ ventilator (Vyaire Medical, Mettawa, IL, USA) with the manufacturer's recommended circuit and nasal prongs (Infant Flow nasal CPAP circuit and prongs, Care Fusion, Dublin, OH, USA) and humidified using an MR850 humidifier (Fisher and Paykel Healthcare, Auckland, New Zealand). The SiPAP device was configured to operate in the biphasic mode, with the low- and high-pressure flow meters set to 8 and 11 L/min, respectively, and the rate and duration of the peak pressure were set to 30 bpm and 1.0 s, respectively. However, an abnormal respiratory pattern indicative of JBS was observed on the first day of life. During apnea episodes, percutaneous oxygen saturation (SpO_2_) decreases, whereas tachypnea is accompanied by retractions. The fraction of inspired oxygen (FiO_2_) was adjusted to maintain SpO_2_ at ≥ 93%; however, episodes of apnea accompanied by a reduction in SpO_2_ were documented 30-50 times daily. To address the apnea, the provision of caffeine citrate was initiated on day seven and subsequently maintained; however, no immediate improvement in symptoms was noted. Consequently, on day eight, respiratory support was transitioned to use of the nCPAP Driver CNO^®^ (Medin Medical Innovations GmbH, Puchheim, Germany), equipped with an MEdiTRIG pressure trigger, in the SNIPPV mode. The SNIPPV mode parameters were configured as follows: base flow rate of 8 L/min, inspiratory flow rate of an additional 3 L/min, push ventilation rate of 20 cycles/min, inspiratory time of 0.5 s, and apnea duration of 5 s. During episodes of apnea, the apnea push is applied; during tachypnea, the trigger push is synchronized with spontaneous breathing, effectively stabilizing the respiratory condition. Notably, following the initiation of CNO, there was a reduction in the incidence of apnea to five episodes per day. No complications, including nasal trauma, air leaks, or hemodynamic changes, were detected during SNIPPV.

Subsequently, given a persistence of the distinctive respiratory pattern associated with JBS, the patient continued to require SNIPPV. Nevertheless, owing to the unavailability of a home ventilator and a nasal mask capable of operating in SNIPPV mode for infants in Japan, tracheostomy was performed on day 82. Subsequently, the patient was managed with synchronized intermittent mandatory ventilation followed by pressure support ventilation on a home ventilator and was discharged on day 198. The patient, now aged one year, continues to require ventilator support.

## Discussion

In this report, we describe the utility of SNIPPV in the respiratory management of abnormal breathing patterns associated with JBS. A number of previous studies have similarly established the efficacy of SNIPPV compared with other ventilation strategies, such as nasal continuous positive airway pressure (NCPAP) and conventional ventilation (CV). Notably, compared with these latter two modes, SNIPPV offers several advantages in reducing the work of breathing, preventing bronchopulmonary dysplasia, shortening the intubation duration, reducing the duration of parenteral nutrition and hospitalization, and treating premature apnea [12-15]. The distinctive respiratory pattern associated with JBS is characterized by alternating episodes of tachypnea and apnea. A number of investigators have deduced that ventilatory abnormalities in neonates with JBS can probably be ascribed to structural defects in the brainstem, particularly those involving the pontine and medullary centers that control respiration [16-18]. Sustained respiratory support is imperative in the absence of a definitive cure for JBS. We suggest that the effective management of SNIPPV in neonates with JBS is attributable to its ability to detect spontaneous breathing during tachypnea via a pressure trigger, synchronizing respiratory support with the patient's breath. In the absence of spontaneous breathing, the backup ventilation feature of SNIPPV maintains ventilation.

The application of SNIPPV with the MEdiTRIG pressure trigger system in neonates has significant clinical implications. Previously, various randomized studies evaluating SNIPPV used pneumatic capsules to detect abdominal movements [7,19,20]. However, ventilators that use these capsules are no longer commercially available. Recently, a non-invasive, Neurally Adjusted Ventilator Assist (NIV-NAVA) device was developed as a variant of SNIPPV and demonstrated superior synchronization. Nevertheless, NIV-NAVA has the disadvantage of requiring the use of a full-featured mechanical ventilator, along with the insertion of an additional catheter to sense the electrical activity of the diaphragm. The Medin CNO® has the advantage of enabling a high synchronization of support without requiring a conventional ventilator or additional catheter insertion. Its compact design and simplified interface offer enhanced convenience and reduced resource consumption, whilst preserving the physiological benefits of SNIPPV. To the best of our knowledge, this is the first study to document the efficacy of SNIPPV with the MEdiTRIG pressure trigger system in the treatment of non-preterm newborns. In addition to premature infants, we believe that this system would provide effective non-invasive respiratory support for neonatal conditions characterized by differing degrees of respiratory disorders, including hypoxic-ischemic encephalopathy, chromosomal disorders, and neuromuscular diseases.

Despite the valuable insights gained in this case study, it does have some limitations. First, the MEdiTRIG pressure trigger system demonstrated an improvement in respiratory condition in this case; however, quantitative and objective data were not available. Further study on the application of this system in neonates is required. Next, although it has previously been reported that the administration of caffeine is effective in managing apnea in patients with JBS [21], in the present case, we found that the short-term administration of caffeine had no evident effects with regards to ameliorating the symptoms. Nevertheless, it is conceivable that longer-term administration may contribute to observable improvements in respiratory dysfunction. Finally, although respiratory management during the neonatal period was successfully achieved using the Median CNO® system (Medin Medical Innovations GmbH, Puchheim, Germany), tracheostomy was ultimately necessary for subsequent home medical care of this patient. Future research should assess the long-term efficacy and safety, as well as home-use adaptations, of SNPPV for infants diagnosed with JBS.

## Conclusions

Patients diagnosed with JBS frequently present with respiratory irregularities, particularly during infancy, and for such cases, it is imperative to implement respiratory support strategies that are tailored to atypical breathing patterns associated with high rates of mortality. In this report, we describe the utility of the SNIPPV mode of the MEdiTRIG pressure trigger system for the respiratory management of abnormal breathing patterns associated with JBS. This approach offers the advantage of enabling high synchronization without requiring the use of a conventional ventilator or additional catheter insertion. Our findings indicate that this system may be effective in managing neonatal conditions characterized by different degrees of respiratory disorders, such as JBS, leading to improved clinical outcomes in this patient population.

## References

[1] Joubert M, Eisenring JJ, Robb JP, Andermann F (1969). Familial agenesis of the cerebellar vermis: a syndrome of episodic hyperpnea, abnormal eye movements, ataxia, and retardation. Neurology.

[2] Romani M, Micalizzi A, Valente EM (2013). Joubert syndrome: congenital cerebellar ataxia with the molar tooth. Lancet Neurol.

[3] Poretti A, Boltshauser E, Valente EM (2014). The molar tooth sign is pathognomonic for Joubert syndrome!. Pediatr Neurol.

[4] Coene KL, Roepman R, Doherty D (2009). OFD1 is mutated in X-linked Joubert syndrome and interacts with LCA5-encoded lebercilin. Am J Hum Genet.

[5] Boltshauser E, Isler W (1977). Joubert syndrome: episodic hyperpnea, abnormal eye movements, retardation and ataxia, associated with dysplasia of the cerebellar vermis. Neuropediatrics.

[6] Dempsey JC, Phelps IG, Bachmann-Gagescu R, Glass IA, Tully HM, Doherty D (2017). Mortality in Joubert syndrome. Am J Med Genet Part A.

[7] Khalaf MN, Brodsky N, Hurley J, Bhandari V (2001). A prospective randomized, controlled trial comparing synchronized nasal intermittent positive pressure ventilation versus nasal continuous positive airway pressure as modes of extubation. Pediatrics.

[8] Bottino R, Pontiggia F, Ricci C (2018). Nasal high-frequency oscillatory ventilation and CO(2) removal: a randomized controlled crossover trial. Pediatr Pulmonol.

[9] Bordessoule A, Moreira A, Felice Civitillo C, Combescure C, Polito A, Rimensberger PC (2021). Comparison of inspiratory effort with three variable-flow nasal continuous positive airway pressure devices in preterm infants: a cross-over study. Arch Dis Child Fetal Neonatal Ed.

[10] Apgar V, Holaday DA, James LS, Weisbrot M, Berrien C (1958). Evaluation of the newborn infant-second report. J Am Med Assoc.

[11] Chen L, Zhao MF, Deng HW (2025). A novel pathogenic splicing nutation of OFD1 is responsible for a boy with Joubert syndrome exhibiting orofaciodigital spectrum anomalies, polydactyly and retinitis pigmentosa. Pharmgenomics Pers Med.

[12] Aghai ZH, Saslow JG, Nakhla T (2006). Synchronized nasal intermittent positive pressure ventilation (SNIPPV) decreases work of breathing (WOB) in premature infants with respiratory distress syndrome (RDS) compared to nasal continuous positive airway pressure (NCPAP). Pediatr Pulmonol.

[13] Bhandari V, Gavino RG, Nedrelow JH, Pallela P, Salvador A, Ehrenkranz RA, Brodsky NL (2007). A randomized controlled trial of synchronized nasal intermittent positive pressure ventilation in RDS. J Perinatol.

[14] Santin R, Brodsky N, Bhandari V (2004). A prospective observational pilot study of synchronized nasal intermittent positive pressure ventilation (SNIPPV) as a primary mode of ventilation in infants≥ 28 weeks with respiratory distress syndrome (RDS). J Perinatol.

[15] Gizzi C, Montecchia F, Panetta V (2015). Is synchronised NIPPV more effective than NIPPV and NCPAP in treating apnoea of prematurity (AOP)? A randomised cross-over trial. Arch Dis Child Fetal Neonatal Ed.

[16] Yachnis AT, Rorke LB (1999). Neuropathology of Joubert syndrome. J Child Neurol.

[17] Sztriha L, Al-Gazali LI, Aithala GR, Nork M (1999). Joubert’s syndrome: new cases and review of clinicopathologic correlation. Pediatr Neurol.

[18] Wolfe L, Lakadamyali H, Mutlu GM (2010). Joubert syndrome associated with severe central sleep apnea. J Clin Sleep Med.

[19] Barrington KJ, Bull D, Finer NN (2001). Randomized trial of nasal synchronized intermittent mandatory ventilation compared with continuous positive airway pressure after extubation of very low birth weight infants. Pediatrics.

[20] Friedlich P, Lecart C, Posen R, Ramicone E, Chan L, Ramanathan R (1999). A randomized trial of nasopharyngeal-synchronized intermittent mandatory ventilation versus nasopharyngeal continuous positive airway pressure in very low birth weight infants after extubation. J Perinatol.

[21] Vodopich DJ, Gordon GJ (2004). Anesthetic management in Joubert syndrome. Paediatr Anaesth.

